# Nephrectomy Delay of More than 10 Weeks from Diagnosis Is Associated with Decreased Overall Survival in pT3 RCC

**DOI:** 10.15586/jkcvhl.v8i2.125

**Published:** 2021-06-14

**Authors:** Jiping Zeng, Ken Batai, Benjamin R. Lee

**Affiliations:** Department of Urology, University of Arizona College of Medicine, Tucson, AZ, USA

**Keywords:** nephrectomy, overall survival, renal cell carcinoma (RCC), surgical wait time (SWT)

## Abstract

In this study, we aimed to evaluate the impact of surgical wait time (SWT) on outcomes of patients with renal cell carcinoma (RCC), and to investigate risk factors associated with prolonged SWT. Using the National Cancer Database, we retrospectively reviewed the records of patients with pT3 RCC treated with radical or partial nephrectomy between 2004 and 2014. The cohort was divided based on SWT. The primary outcome was 5-year overall survival (OS). Logistic regression analysis was used to investigate the risk factors associated with delayed surgery. Cox proportional hazards models were fitted to assess relations between SWT and 5-year OS after adjusting for confounding factors. A total of 22,653 patients were included in the analysis. Patients with SWT > 10 weeks had higher occurrence of upstaging. Using logistic regression, we found that female patients, African-American or Spanish origin patients, treatment in academic or integrated network cancer center, lack of insurance, median household income of <$38,000, and the Charlson–Deyo score of ≥1 were more likely to have prolonged SWT. SWT > 10 weeks was associated with decreased 5-year OS (hazard ratio [HR], 1.24; 95% confidence interval [CI], 1.15–1.33). This risk was not markedly attenuated after adjusting for confounding variables, including age, gender, race, insurance status, Charlson–Deyo score, tumor size, and surgical margin status (adjusted HR, 1.13; 95% CI, 1.04–1.24). In conclusion, the vast majority of patients underwent surgery within 10 weeks. There is a statistically significant trend of increasing SWT over the study period. SWT > 10 weeks is associated with decreased 5-year OS.

## Introduction

Advanced stage renal cell carcinoma (RCC) remains as one of the most lethal urological cancers in spite of advancements made in the diagnosis and treatment over the last decade (1). In the United States, there are approximately 74,000 new cases and almost 15,000 deaths from RCC each year (2). Partial nephrectomy or radical nephrectomy with curative intent is the treatment of choice for RCC. Treatment delay, sometimes in months, occurs when there is increase in surgeon case load, insurance issues, and preoperative optimization of medical comorbidities. Globally, the surgery wait time (SWT) for cancer treatment has increased over the last decade (3) combined with an aging population and retirement of physicians.

From a patient’s perspective, prolonged waiting for cancer treatment causes anxiety and distress, but current published medical literature has failed to provide a definitive conclusion or insight into change in outcomes resulting from prolonged SWT (4, 5). One of the most frequent questions that a surgeon or the scheduler encounters in clinic is whether the prolonged SWT has a negative impact on oncologic outcomes. It has been shown in the case of muscle invasive bladder cancer that radical cystectomy delayed for more than 12 weeks results in increased risk of disease-specific and all-cause mortality (6). Similarly, prolonged time to surgery confers lower overall and disease-specific survival in breast cancer, and a shortened delay is associated with benefits comparable to some standard therapies (7).

To date, few reports have investigated the effect of prolonged SWT on oncologic outcomes following nephrectomy. In this study, we used the National Cancer Database (NCDB) to investigate the impact of delayed surgery. We specifically examined the pT3 group as this represents advanced stage tumor that has invaded locally, and it could be benefited the most from timely treatment.

## Materials and Methods

Using the NCDB, we retrospectively reviewed the records of adult patients (aged ≥18 years) with pT3 RCC treated with radical nephrectomy or nephron sparing surgery between 2004 and 2014. The NCDB is a hospital-based clinical cancer registry established in 1989 that collects data from more than 1500 hospitals in the United States, capturing more than 70% of all newly diagnosed cancers (8, 9). The following histology codes were used to select the patients: 8255, 8260, 8310, 8316, 8317, 8318, and 8319. Patients whose final pathology reports presented benign tumors were excluded from the study. The SWT in days was derived from “Definitive Surgical Procedure, Days from Dx.” The diagnosis of RCC is based on preoperative cross-sectional imaging. A small group of patients had a 0 day interval between diagnosis and surgery, and were excluded from the analysis as this group likely represented referral from outside hospital and such SWT was incorrect. Patients with metastatic disease were also excluded from the study.

Follow-up time in months was calculated using last date of contact or date of death since definitive surgery. The primary outcome was 5-year overall survival (OS), which was calculated using vital status (alive or death) and last date of contact since definitive surgery. We used Cox proportional-hazards model to analyze the effect of SWT in weeks, and found a significant difference in the 5-year OS at a time of 10 weeks. This time was used for subsequent analysis.

Patient demographics and tumor characteristics were summarized and compared between those who had surgery within and after 10 weeks. We then performed trend analysis of SWT for the study period using the Jonckheere–Terpstra test. Binominal logistic regression analysis was used to investigate the risk factors associated with SWT > 10 weeks. We used Chi-square test to compare perioperative outcomes between the two groups, including surgical margin status, 30-day mortality, 90-day mortality, and readmission within 30 days of discharge. We used the Mann–Whitney U-test to compare the length of stay. Patients whose preoperative clinical staging was cT1 or cT2 were captured and marked as upstaging. The rate of upstaging was compared in two groups. Logistic regression analysis was carried out to investigate whether SWT > 10 weeks was associated with risk of upstaging after adjusting for confounding factor, tumor size. Next, Cox proportional hazards models were fitted to assess relations between SWT and 5-year OS, adjusting for patient demographics (age, gender, race, and insurance status), comorbidities, and tumor characteristics (Charlson–Deyo score, tumor size, and surgical margin status).

All statistical analyses were performed using SPSS version 25.0 (SPSS, Inc., Chicago, IL). P < 0.05 was considered significant.

## Results

A total of 22,653 patients from 2004 to 2014 were included in the analysis. The median follow-up time was 31 months. The median interval between diagnosis and definitive surgery was 29 days, and 85.3% of the patients underwent nephrectomy within 10 weeks of diagnosis. Patient demographics and tumor characteristics are summarized in Table 1.

**Table 1:** Patient demographics and tumor characteristics.

	P-value	SWT < 10 weeks	SWT > 10 weeks
Age (years) <40 40–60 >60	<0.01	420 (2.2%) 7653 (39.6%) 11,240 (58.2%)	55 (1.6%) 1093 (32.7%) 2192 (65.6%)
Gender Male, N (%) Female, N (%)	0.083	13,222 (68.5%) 6091 (31.5)	2337 (70%) 1003 (30%)
Race White Black Other	<0.01	17,244 (89.3%) 1248 (6.5%) 821 (4.3%)	2852 (85.4%) 334 (10.0%) 154 (4.6%)
Spanish origin No Yes Unknown	<0.01	17,118 (88.6%) 1141 (5.9%) 1054 (5.5%)	2898 (86.8%) 275 (8.2%) 167 (5.0%)
Facility type Community Academic Unknown	<0.01	7757 (40.2%) 11,136 (57.7%) 420 (2.2%)	1015 (30.4%) 2270 (68%) 55 (1.6%)
Insurance status Not insured Insured Unknown	<0.01	684 (3.5%) 18,391 (95.2%) 238 (1.2%)	144 (4.3%) 3151 (94.3%) 45 (1.3%)
Income <38,000 >38,000	<0.01	3143 (16.3%) 15,923 (82.4%)	676 (20.2%) 2627 (78.7%)
Charlson–Deyo score 0 1 2 or more	<0.01	15,472 (68.3%) 5392 (23.8%) 1789 (7.9%)	2081 (62.3%) 886 (26.5%) 373 (11.2%)
Urban/rural Metro Urban Rural	0.073	14,823 (76.8%) 3363 (17.4%) 498 (2.6%)	2596 (77.7%) 559 (16.7%) 65 (1.9%)
Great circle distance ≤100 miles >100 miles Unknown	0.67	17,298 (89.6%) 1762 (9.1%) 253 (1.3%)	2989 (89.5%) 313 (9.4%) 88 (1.1%)
Grade Well differentiated Moderately differentiated Poorly differentiated Undifferentiated Unknown	<0.01	428 (2.2) 4627 (24%) 7520 (38.9%) 3717 (19.2%) 3021 (15.6%)	103 (3.1%) 912 (27.3%) 1230 (36.8%) 496 (14.9%) 599 (17.9%)
Size of tumor ≤10 cm >10 cm	<0.01	12,770 (66.1%) 6543 (33.9%)	2618 (78.4%) 722 (21.6%)

SWT: surgical wait time.

**Figure 1:**
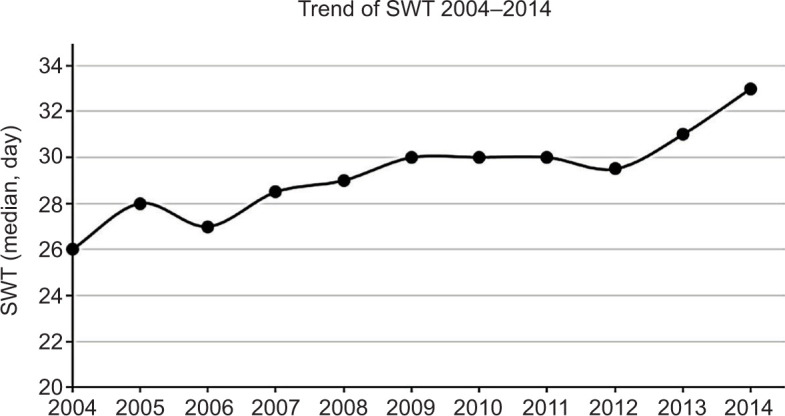
Trend analysis of surgical wait time (SWT) for the study period 2004–2014. Figure demonstrates the median SWT of each year. The Jonckheere–Terpstra test shows a statistically significant trend of increasing SWT from 2004 to 2014.

The Jonckheere–Terpstra test for ordered alternatives showed that there was a statistically significant trend of increasing SWT over the study period (P < 0.001, Figure 1). There was no statistical difference in the length of stay and readmission rate between the two groups (Table 2). Patients (n = 3340) who had a delay of more than 10 weeks had significantly higher occurrence of upstaging (cT1 or cT2 upstaged to pT3) compared to those who underwent surgery within 10 weeks. On logistic regression analysis, SWT within 10 weeks was associated with less chance of upstaging after adjusting for tumor size (adjusted odds ratio [OR], 0.71; 95% CI, 0.65–0.78). In all pT3 patients, the 30-day mortality was 2% (n = 447), while 90-day mortality was 5.3% (n = 1208). There was no significant difference in 30-day or 90-day mortality rates between the two groups. Interestingly, patient who underwent nephrectomy within 10 weeks had a positive surgical margin rate of 16.8%, which was significantly higher than that of those with SWT > 10 weeks. No significant difference was noted in 30-day readmission rate between the two groups.

**Table 2:** Treatment outcomes by groups.

	P-value	SWT < 10 weeks	SWT > 10 weeks
Upstaged to pT3	<0.01	6601 (34.2%)	1456 (43.6%)
Surgical margin Negative Positive Unknown	<0.01	15,684 (81.2%) 3237 (16.8%) 392 (2%)	2849 (85.3%) 425 (12.7%) 66 (2%)
LOS (Mean ± Std)	0.064	5.3 ± 5.8 d	5 ± 5.4 days
Readmission Yes No Unknown	0.051	732 (3.8%) 18,293 (94.7%) 288 (1.5%)	146 (4.4%) 3136 (93.9%) 58 (1.7%)
30-Day mortality	0.606	390 (2%)	57 (1.7%)
90-Day mortality	0.659	1051 (9%)	157 (4.7%)

SWT: surgical wait time; LOS: length of stay.

Using logistic regression, we found that female patients, African-American or Spanish origin patients, treatment in academic or integrated network cancer center, lack of insurance, median household income of less than $38,000, and the Charlson–Deyo score of ≥1 were more likely to have a delay of more than 10 weeks (Table 3). SWT > 10 weeks was found to be associated with decreased 5-year OS compared with patients who had definitive surgery within 10 weeks (hazard ratio [HR], 1.24; 95% CI, 1.15–1.33). This risk was not markedly attenuated after adjusting for confounding variables, including age, gender, race, insurance status, Charlson–Deyo score, tumor size, and surgical margin status (adjusted HR, 1.13; 95% CI, 1.04–1.24; Table 4).

**Table 3:** Logistic regression analysis for surgical wait time (SWT) > 10 weeks.

	OR	95% CI	P-value
Age (years) <40 40–60 >60	Ref. 0.82 1.18	0.61–1.11 0.87–1.59	0.21 0.29
Gender Male, N (%) Female, N (%)	Ref. 0.91	0.83–0.98	0.018
Race White Black Other	Ref. 1.54 1.05	1.34–1.76 0.87–1.26	<0.01 0.57
Spanish origin No Yes Unknown	Ref. 1.44 0.95	1.27–1.70 0.80–1.13	<0.01 0.60
Facility type Community Academic Unknown	Ref. 1.62 1.91	1.49–1.76 1.39–2.63	<0.01 <0.01
Insurance status Not insured Insured Unknown	Ref. 0.69	0.563–0.84	<0.01
Income <38,000 >38,000	Ref. 0.81	0.74–0.90	<0.01
Charlson–Deyo score 0 1 2 or more	Ref. 1.25 1.63	1.14–1.36 1.43–1.85	<0.01 <0.01
Urban/rural Metro Urban Rural	Ref. 0.95 0.77	0.85–1.10 0.58–1.02	0.36 0.06
Great circle distance ≤100 miles >100 miles	Ref. 0.96	0.84–1.10	0.54

OR: odds ratio; 95% CI: 95% confidence interval.

**Table 4:** Multivariate cox regression for 5-year overall survival.

	HR	95% CI	P-value
Age (years) <40 40–60 >60	Ref. 1.07 1.31	0.93–1.24 1.14–1.51	0.36 <0.01
Gender Male, N (%) Female, N (%)	Ref. 1.02	0.98–1.06	0.33
Race White Black Other	Ref. 1.13 0.83	1.05–1.21 0.75–0.92	<0.01 <0.01
Spanish origin No Yes Unknown	Ref. 0.81 1.00	0.75–0.89 0.93–1.08	<0.01 0.97
Insurance status Not insured Insured Unknown	Ref. 0.98 1.02	0.88–1.09 0.84–1.24	0.75 0.82
Charlson–Deyo score 0 1 2 or more	Ref. 1.07 1.34	1.02–1.12 1.25–1.43	<0.01 <0.01
Tumor size ≤10 cm >10 cm	Ref. 0.64	0.62–0.66	<0.01
Surgical margin Negative Positive Unknown	Ref. 1.88 1.73	1.79–1.96 1.54–1.94	<0.01 <0.01
SWT ≤10 weeks >10 weeks	Ref. 1.13	1.04–1.24	<0.01

HR: hazard ratio; SWT: surgical wait time; 95% CI: 95% confidence interval.

## Discussion

Prolonged SWT has significant impact on the psychological wellbeing of cancer patients (4). In the current study, we demonstrated that delayed nephrectomy for more than 10 weeks from the time of diagnosis is associated with decreased 5-year OS in a group of pT3 RCC patients within a national database. Additionally, we found that female patients, African-American or Spanish origin patients, treatment in academic or integrated network cancer center, lack of insurance, median household of income less than $38,000, and the Charlson–Deyo score of ≥1 are more likely to have a delayed definitive surgery.

The current recommendation for treatment of RCC greater than 4 cm in size is nephrectomy. This could be performed through either open approach or laparoscopic or robotic approach. With a wide adoption of da Vinci Surgical System, radical nephrectomy and partial nephrectomy are performed more routinely via robotic or laparoscopic approach in tertiary care centers. Nevertheless, most surgeons who perform robotic surgical procedures are assigned block time because of the limited number of robotic systems available in most hospitals. A recent study has shown that this causes a bottleneck effect and increase in the number of patients with considerably lengthy waiting time for surgery (10). This factor is not included in our study, but it may affect the decision-making of surgical approach and ultimately SWT.

In 2006, Canadian Surgical Wait Times (SWAT) Initiative proposed a recommended maximum wait time of <90 days for patients with T1a RCC and <28 days for patients with symptomatic tumors (11). However, previous reports have shown that small renal masses tend to grow slowly with linear growth rate of 0.13–0.7 cm/year, and these tumors can be safely checked (12, 13). Active surveillance remains a reasonable alternative to surgery for these tumors for elderly or comorbid ill patients. For T1a RCC, the interval between diagnosis and nephrectomy has been reported to be more than 2 years in literature (14, 15). In a group of 82 patients with a median tumor size of 2 cm, surgical management at 6–97 months has not resulted in limitation of treatment options or a high risk of disease progression (16).

Kim et al. reported an analysis on delaying radical nephrectomy for stage II or higher RCC (17). SWT of 1–3 months was not an independent predictor of pathological upstaging, recurrence-free survival, or cancer-specific survival (CSS). On subgroup analysis by TNM staging system (cT2NxcM0 and cT3-4NxcM0), SWT of 1–3 months was not an independent predictor of pathological upstaging and was not associated with poor recurrence-free survival (RFS) or CSS. However, patients with SWT > 3 months were excluded from the analysis. In a single institution analysis, a group of 655 patients with 6.4 ± 4.4-cm renal tumors who underwent partial or radical nephrectomy were reviewed retrospectively: 64.1% and 94.3% of the patients had surgery within 30 days and 3 months, respectively. OS and disease-specific survival were not affected by surgical wait time regardless of how time was analyzed. Interestingly, in univariate analysis, 5-year recurrence-free survival was poorer in patients undergoing surgery within 1 month, likely secondary to larger, and more sinister-appearing renal masses being pushed up in the operating schedule (18). Again, this study has not included the patients who underwent surgery in more than 3 months. In another single institutional analysis, patients with a tumor size of 5.5 ± 3.45 cm were included. SWT stage for stage was: clinical T1 at 57.12 days, clinical T2 at 36.8 days, and clinical T3 and T4 at 30.32 days. There was no statistically significant evidence for upstaging or progression during the waiting period (19). Mano et al. found that for patients with renal mass of >4 cm, SWT was not associated with disease upstaging, recurrence, or CSS (20). Longer SWT was associated with decreased OS. Older age, non-white race, higher BMI, higher American Society of Anesthesiologists (ASA) and Charlson comorbidity index (CCI) scores, incidental presentation, smaller tumor size, non-clear cell histology, and treatment with partial nephrectomy were significantly associated with SWT > 3 months on univariate analysis (20). In our study, we found that treatment in academic or integrated network cancer center was associated with delayed surgery. However, we suspect that this was due to referral patterns common with tertiary referral centers because of increasing complexity of the case which mandates multidisciplinary workup in medical center and potential cardiac evaluation, in addition to case load contribution. Indeed, patient having the Charlson–Deyo score of 1 or ≥2 and an HR of 1.25 and 1.63 would have an SWT > 10 weeks in our analysis. This delay in surgery reflects the preoperative workup and optimization of comorbidities that take up time.

This study has several limitations. First, this is a retrospective review of a large national database that may contain selection bias not unveiled by the current analysis (21, 22). Selection bias exists in clinical practice and surgeons tend to schedule patient with larger tumor for earlier OR dates if possible. Second, the diagnosis of RCC is made with cross-sectional imaging unlike bladder and prostate cancers, which are diagnosed with biopsy and have a definitive diagnosis date; the interval between surgery and diagnosis may have been less accurate in this regard. In this database, we also noticed a portion of patients who had 0 day interval between diagnosis and surgery. This likely results from reimaging of the patient on the day of the surgery when progression is suspected, or results from the patient referral who had data missing in the initial encounter and has surgery along with outside imaging uploaded on the same day. We excluded this portion of patients from the analysis; however, this may represent a bias in statistics. Lastly, although the analysis is adjusted for confounding factors, this database provides OS not ideal for outcome analysis. CSS along with recurrence data would provide more directed analysis. The future efforts must be directed to investigate the impact of delayed surgery in a prospective manner.

## Conclusion

In the current study, we used the NCDB and found that the vast majority of pT3 patients underwent radical or partial nephrectomy within 10 weeks. Delaying definitive surgery for more than 10 weeks is associated with decreased 5-year OS but not with an increased risk of 30- or 90-day mortality. SWT > 10 weeks is associated with tumor upstaging. Additionally, we found that female patients, African-American or Spanish origin patients, treatment in academic or integrated network cancer center, lack of insurance, median household income of less than $38,000, and the Charlson–Deyo score of ≥1 are more likely to have delayed definitive surgery. Surgery scheduling is a complex issue and this study adds to the current consideration along with patient comorbidities and tumor characteristics.
